# 9-oxo-ODAs suppresses the proliferation of human cervical cancer cells through the inhibition of CDKs and HPV oncoproteins

**DOI:** 10.1038/s41598-023-44365-3

**Published:** 2023-11-06

**Authors:** Kazumasa Mogi, Yoshihiro Koya, Masato Yoshihara, Mai Sugiyama, Rika Miki, Emiri Miyamoto, Hiroki Fujimoto, Kazuhisa Kitami, Shohei Iyoshi, Sho Tano, Kaname Uno, Satoshi Tamauchi, Akira Yokoi, Yusuke Shimizu, Yoshiki Ikeda, Nobuhisa Yoshikawa, Kaoru Niimi, Yoshihiko Yamakita, Hiroyuki Tomita, Kiyosumi Shibata, Akihiro Nawa, Yutaka Tomoda, Hiroaki Kajiyama

**Affiliations:** 1 Department of Obstetrics and Gynecology, Nagoya University Graduate School of Medicine, Tsuruma-Cho 65, Showa-Ku, Nagoya, Aichi Japan; 2 Bell Research Center Obstetrics and Gynecology Academic Research & Industrial - Academia Collaboration Nagoya University Graduate School of Medicine, Nagoya University, Tsuruma-Cho 65, Showa-Ku, Nagoya, Aichi Japan; 3 Discipline of Obstetrics and Gynaecology, Adelaide Medical School, Robinson Research Institute, University of Adelaide, Adelaide, Australia; 4 Department of Obstetrics and Gynecology, Kitazato University, Kanagawa, Japan; 5 Spemann Graduate School of Biology and Medicine, University of Freiburg, Breisgau, Germany; 6 Institute for Advanced Research, Nagoya University, Nagoya, Aichi Japan; 7 Division of Clinical Genetics, Lund University, Lund, Sweden; 8 Department of Tumor Pathology, Gifu University Graduate School of Medicine, Gifu, Japan; 9 Department of Obstetrics and Gynecology, Bantane Hospital, Fujita Health University, Nagoya, Aichi Japan; 10 Tomoda Ladies Clinic, Chita, Aichi Japan

**Keywords:** Drug development, Cancer therapy, Cancer, Gynaecological cancer, Cervical cancer

## Abstract

Mucosal human papillomavirus (HPV) subtypes 16 and 18 are causative agents of cervical cancer, a leading cause of cancer-related deaths among women worldwide. In Japan, eggplant calyx is a folk remedy used to treat common warts. 9-oxo-(10E,12E)-octadecadienoic acid, isolated from eggplant calyx, may have antitumor effects. This study investigated the antitumor effects of 9-oxo-(10E, 12Z)-octadecadienoic acid and 9-oxo-(10E,12E)-octadecadienoic acid (9-oxo-ODAs) on human cervical cancer cells. 9-oxo-ODAs suppressed the proliferation of human cervical cancer cell lines (HeLa, and SiHa) in a concentration-dependent manner (IC50 = 25–50 µM). FCM analysis revealed that 9-oxo-ODAs induced apoptosis. Transcriptome, proteomics, and enrichment analyses revealed that treatment with 9-oxo-ODAs significantly altered the cell cycle and p53 pathways and decreased cyclin-dependent kinase 1 (CDK1) protein expression. Real-time PCR analysis demonstrated that 9-oxo-ODAs reduced *CDK1* mRNA expression in a concentration-dependent manner. In vitro, 9-oxo-ODAs reduced the HPV oncoprotein expression. In ex vivo human cervical cancer tissues, 9-oxo-ODAs decreased CDK1 expression and increased cleaved caspase 3, an apoptosis marker. Further, 9-oxo-ODAs showed the potential to suppressed metastatic formation and growth of cervical cancer in vivo. These findings suggest that 9-oxo-ODAs induce cell cycle arrest and apoptosis in HPV-positive human cervical cancer cells, and this process involves CDK1. Consequently, 9-oxo-ODAs may be potential therapeutic agents for cervical cancer.

## Introduction

Cervical cancer is the fourth most common cancer among women, accounting for an estimated 604,000 cases and 342,000 deaths worldwide in 2020^1,2^. In Japan, the number of new cases was 11,012 in 2017, and number of deaths was 2921 in 2019. Notably, deaths due to cervical cancer have been gradually increasing since 2000 (https://ganjoho.jp/reg_stat/statistics/stat/summary.html). Cervical cancer is caused by human papillomavirus (HPV), which is detected in 99.7% of cervical cancers^3^. In the 1990s, epidemiological studies using molecular biology techniques revealed that HPV infections were involved in the development of cervical cancer^4^. Vaccines have since been developed and are highly effective for preventing this disease, with widespread vaccination rates globally^5^. However, the introduction of vaccinations in Japan has been delayed. The number of cervical cancer-related deaths in less-developed countries remains one of the highest worldwide due to the lack of vaccination opportunities and high incidence of cervical cancer.

For the HPV oncogenic mechanism, E6 and E7 are important oncoproteins. E6 persists in a stable complex with E6AP, a ubiquitin-protein ligase, which promotes p53 degradation, contributing to carcinogenesis^6–9^. E7 also degrades and inactivates pRB, continuously activating the E2F transcription factor and promoting the cell cycle^10,11^. Notably, these oncoproteins E6 and E7 are reported to be required for the growth and survival of HPV-positive cervical cancer strains^12^. Although these oncoproteins have been well reported to function in carcinogenesis and progression, therapies targeting these oncoproteins and mechanisms are not yet clinically available.

Anticancer drugs developed from natural compounds, such as paclitaxel, vincristine, camptothecin, doxorubicin, and eribulin, are currently used for the treatment of many cancers.

It was recently reported that petasin, identified from Petasites japonicus, inhibits mitochondrial electron transport chain complex I (ETCC1), an essential component of cancer metabolism, and exhibits antitumor effects^13^. In gynecologic oncology, β-escin extracted from the seed of the horse chestnut, *Aesculus hippocastanum L.*, inhibits ovarian cancer metastasis by targeting cancer and stromal cells and suppressing the production of ECM and HIF1α-targeted proteins, lactate dehydrogenase A, and hexokinase 2 in omental tumors^14,15^. These findings support the potential of using natural compounds for drug development.

*Eggplants*, the fruits of *Solanum melongena L. (Solanaceae)*, are popular vegetables in Asia. In Japan, the calyx of eggplants has been used as a folk remedy for common warts caused by human papillomavirus (HPV)^16^. We previously reported that an ethanol extract of dried eggplant calyx reduced ovarian cancer and condyloma^17,18^. The lethal activity of this extract against various human cancer cell lines was examined, and the compound exhibited inhibitory activity against cell growth in human ovarian cancer cell lines. Two cell growth inhibitory components, 9-oxo-(10E, 12Z)-octadecadienoic acid and 9-oxo-(10E, 12E)-octadecadienoic acid (both termed 9-oxo-ODAs), were isolated and identified from this extract using activity-based fractionation^19^.

In this study, we evaluated the growth inhibitory effects of 9-oxo-ODAs in cervical cancer caused by HPV infection. We confirmed the antitumor effects of 9-oxo-ODAs in vitro, identified their mechanisms of action using omics analysis, and verified their effects ex vivo and in vivo.

## Results

### 9-oxo-ODAs inhibit the growth of cervical cancer cell lines in vitro

**Figure 1:**
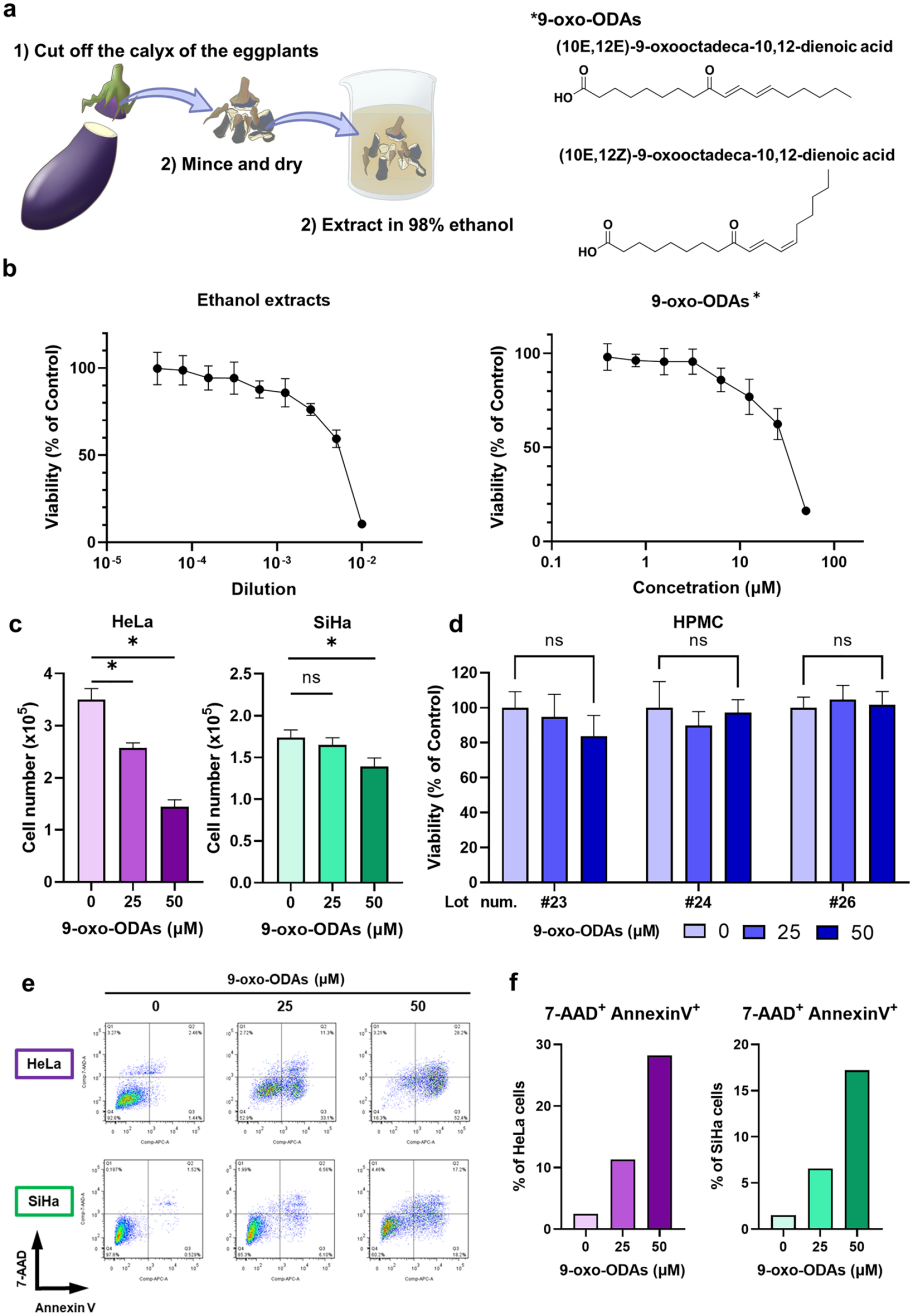
Inhibition of proliferation of cervical cancer cell lines by 9-oxo-ODAs from eggplant. (**a**) Schematic diagram of the process of extracting the active ingredients from an eggplant calyx. The extract contains active ingredients, 9-oxo-ODAs (two isomers: 9-oxo-(10E,12Z)-octadecadienoic acid and 9-oxo-(10E,12E)-octadecadienoic acid). (**b**) Inhibitory effects on HeLa cell proliferation. Left panel: dilution series of the ethanol extract of an eggplant calyx used to treat HeLa cells; right panel: dilution series of 9-oxo-ODAs (active ingredient) used to treat HeLa cells. The viable cell rate was evaluated using WST-8. (**c**) Growth Inhibitory effects of 9-oxo-ODAs on HeLa and SiHa cell growth. The number of viable cells was measured 5 days later by trypan blue exclusion. (**d**) The viable cell rate was measured using the WST-8 assay on day 5 after administration of 9-oxo-ODAs to peritoneal mesothelial cells, with normal cells as a control. (**e**,**f**) Apoptosis analysis of HeLa and SiHa cells treated with 9-oxo-ODAs using flow cytometry.

We initially verified the antitumor effects of an ethanol extract of eggplant calyx and its active compound, 9-oxo-ODAs, on cervical cancer cell lines. The process of ethanol extraction of the active compound from the eggplant calyx and the chemical structure of 9-oxo-ODAs are presented in Fig. 1a. Treatment of the HeLa cervical cancer cell line with ethanol extracts and 9-oxo-ODAs revealed that the ethanol extract reduced the number of viable tumor cells, and 9-oxo-ODAs exerted a similar effect. The IC50 of 9-oxo-ODAs in HeLa cells was 30.532 μM (Fig. 1b).

Administration of 9-oxo-ODAs to HeLa and SiHa cervical cancer cell lines resulted in a decrease in the number of HeLa and SiHa cells in a concentration-dependent manner (Fig. 1c). Human peritoneal mesothelial cells were also treated with 9-oxo-ODAs as normal cells, but no significant effects were observed on the number of viable human peritoneal mesothelial cells (HPMC). These results indicated that 9-oxo-ODAs reduced the viable cell count of cervical cancer cell lines but did not affect normal cells (Fig. 1d). Additionally, 9-oxo-ODAs significantly decreased the number of HeLa, an HPV-infected cell line, but not non-HPV-infected cell lines HSC-2, SCC-25, SAS (Fig. S1). To confirm the mechanism by which 9-oxo-ODAs reduced the number of viable cervical cancer cells, we examined the effects of 9-oxo-ODAs on apoptosis in these cell lines. Using flow cytometry, we measured the number of apoptotic cells labeled with annexin V and 7-AAD in HeLa and SiHa cells treated with 9-oxo-ODAs (Fig. 1e,f). The analysis revealed that 9-oxo-ODAs increased the number of apoptotic cells labeled with annexin V and 7-AAD in a concentration-dependent manner. These results suggest that 9-oxo-ODAs induce apoptosis of cervical cancer cells and inhibit cell proliferation.

### 9-oxo-ODAs alter RNA expression profiles of cervical cancer cell lines

**Figure 2:**
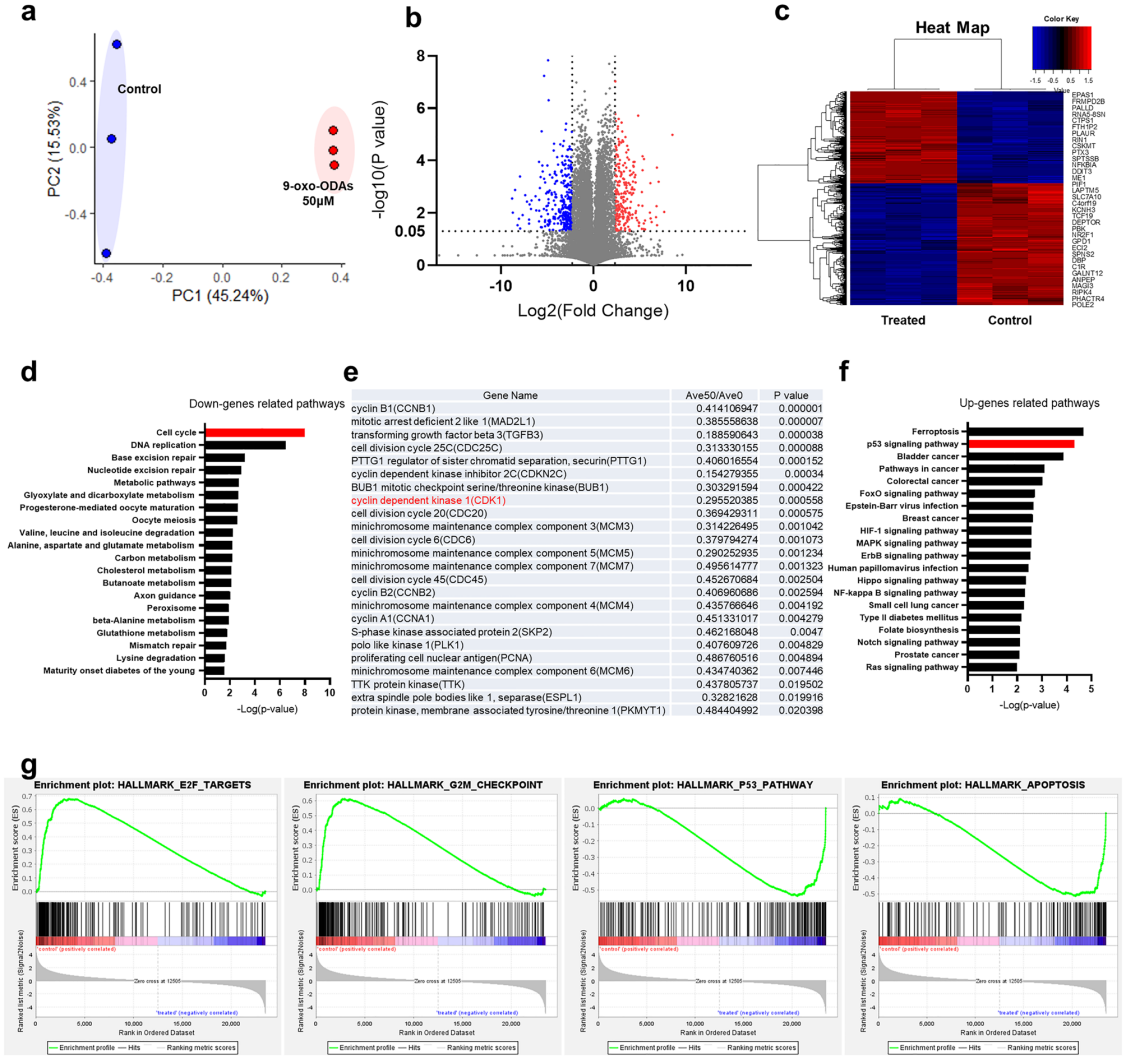
Results of transcriptome and pathway analyses of HeLa cells treated or untreated with 9-oxo-ODAs. (**a**) PCA plots for RNA sequencing. (**b**) Volano plots for RNA sequencing. (**c**) Heat map of genes with significantly altered expression levels. (**d**) DAVID KEGG pathway analysis list associated with downregulated genes for 9-oxo-ODAs vs control. (**e**) List of genes related to cell cycle pathways. The list includes cyclin-dependent kinase 1 (*CDK1*). (**f**) DAVID KEGG pathway analysis list of associated upregulated genes for 9-oxo-ODAs vs control. (**g**) Results of GSEA enrichment analysis and representative pathways.

To determine the mechanisms by which 9-oxo-ODAs induce apoptosis in cervical cancer cells, transcriptome analysis was performed on HeLa cells treated with 9-oxo-ODAs (50 μM) or vehicle (DMSO) (Fig. 2a–c). Furthermore, DAVID enrichment analysis revealed that the cell cycle pathway was significantly altered in the down-regulated gene group (Fig. 2d). Figure 2e shows the list of genes related to the cell cycle pathway. The p53 signaling pathway was significantly altered in the upregulated gene groups (Fig. 2f). Furthermore, GSEA enrichment analysis revealed cell cycle and p53 signaling-related pathways such as “E2F TARGETS,” “G2M CHECKPOINT,” “P53 PATHWAY”, and “APOPTOSIS” (Fig. 2g). Collectively, transcriptome analysis supported that 9-oxo-ODA affects the cell cycle and apoptosis of cervical cancer cells.

### 9-oxo-ODAs modulate protein secretion in a concentration-dependent manner

**Figure 3:**
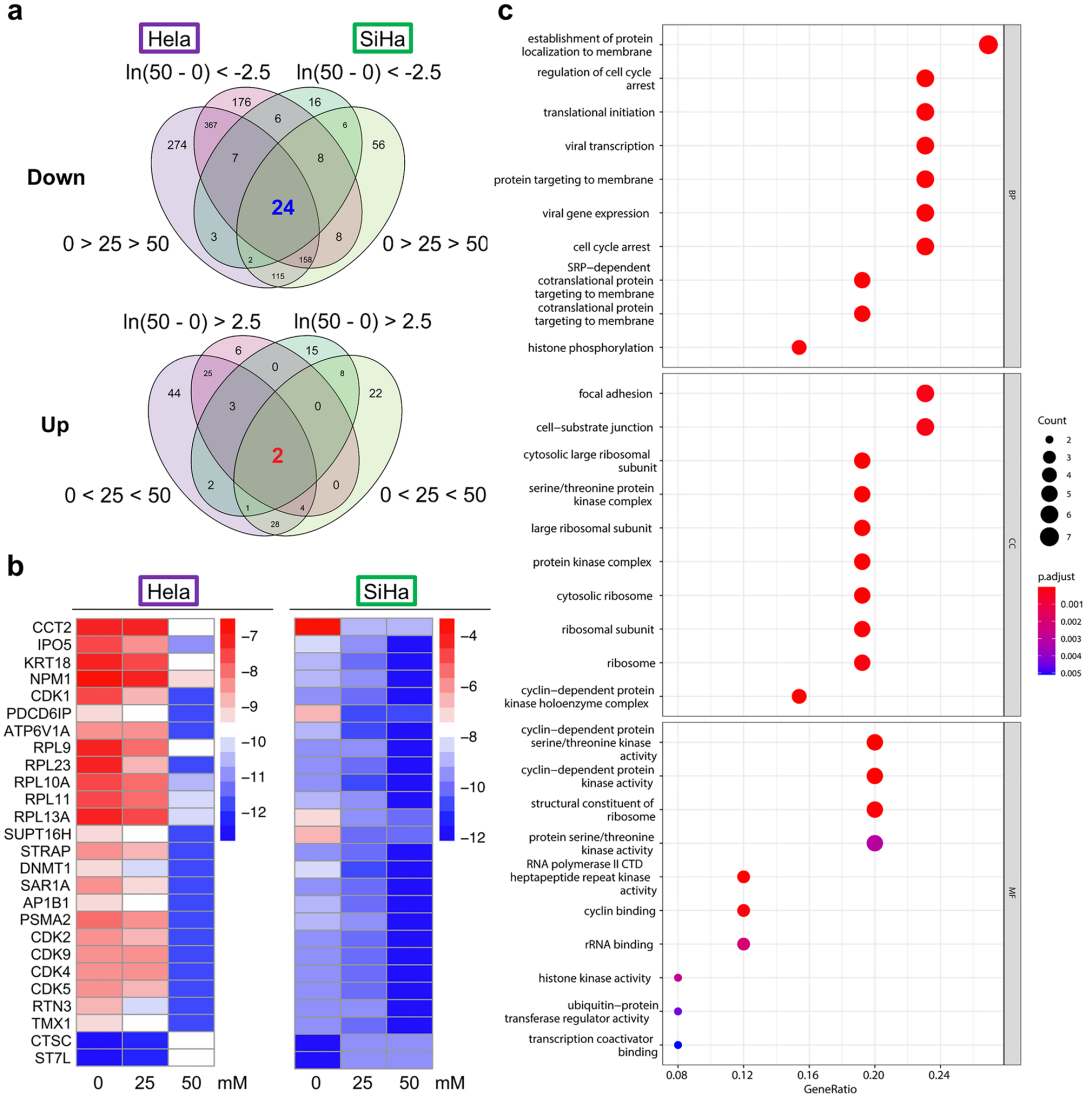
Results of proteomics analysis of HeLa and SiHa cells treated with 9-oxo-ODAs (0, 25, and 50 μM). (**a**) Venn diagram showing the criteria for identifying altered proteins in HeLa and SiHa cells. (**b**) Heat map showing proteins with concentration-dependent changes in both HeLa and SiHa cells. (**c**) Pathways from analysis of identified proteins. Following softwares were used for graphic creation: R (V4.1.1), RStudio (2021.09.0), gplots package (V3.1.3, https://cran.r-project.org/web/packages/gplots/index.html), pheatmap package (https://cran.r-project.org/web/packages/pheatmap/index.html), clusterProfiler package (V4.0.5, https://bioconductor.org/packages/release/bioc/html/clusterProfiler.html).

Next, proteomics analysis was performed on HeLa and Siha cells, which were treated with different concentrations of 9-oxo-ODAs, 0, 25, and 50 μM (Fig. 3a). This proteomics analysis focused on proteins with expression levels that were altered by 9-oxo-ODAs in a concentration-dependent manner. We identified common proteins that were altered in both cell lines.

In total, we identified 24 proteins with a concentration-dependent decrease and 2 proteins with a concentration-dependent increase (Fig. 3b). Enrichment analysis was performed based on the identified proteins (Fig. 3c). Proteome enrichment analysis revealed many cell cycle-related pathways. The results of the omics analyses led us to focus on cyclin-dependent kinase (CDK), which is involved in cell cycle-related pathways and emerged as a candidate in both analyses.

### 9-oxo-ODAs suppress CDK1 and alter the cell cycle

**Figure 4:**
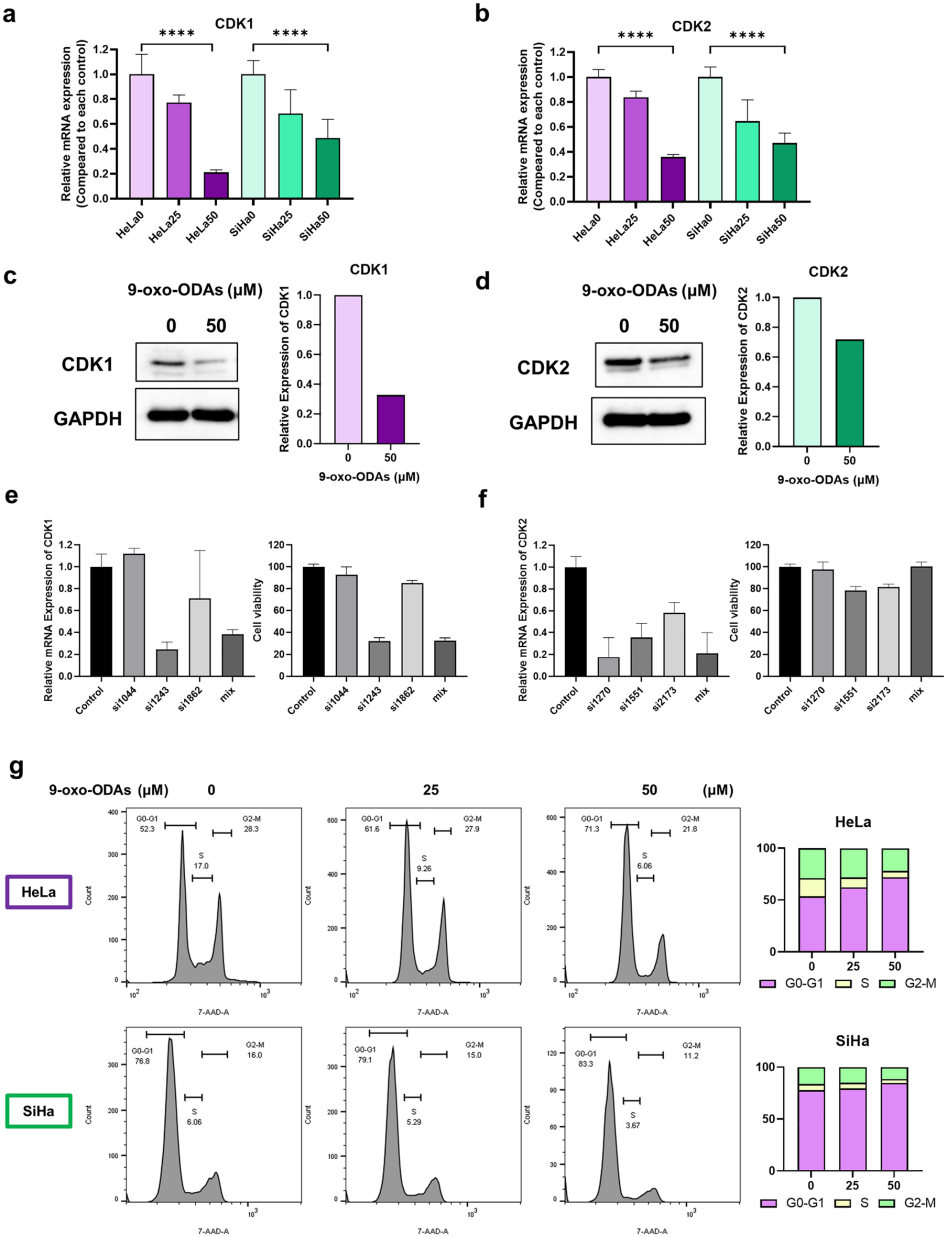
Effects of 9-oxo-ODA treatment on CDK1 and CDK2 expression and cell cycle. (**a, b**) Relative expression levels of (**a**) *CDK1* and (**b**) *CDK2* mRNA in HeLa and SiHa cells treated with 9-oxo-ODAs determined using qPCR. (**c, d**) Immunoblot analysis of (**c**) CDK1 and (**d**) CDK2 proteins in HeLa cells treated with 9-oxo-ODAs. These blots were cut prior to hybridization with antibodies, and the blots are presented in Supplementary Fig. 2. (**e**,**f**) Effects of suppressing (**e**) *CDK1* and (**f**) *CDK2* mRNA expression on HeLa cell proliferative capacity. (**g**) Effects of 9-oxo-ODA treatment on cell cycle in HeLa and SiHa cells determined using flow cytometry.

Based on our results, we focused on CDK1 and CDK2 and analyzed changes in CDK1/CDK2 expression after 9-oxo-ODA treatment. The analysis revealed a decrease in CDK1 and CDK2 mRNA and protein levels (Fig. 4a–d). 9-oxo-ODAs decreased *CDK1* and *CDK2* mRNA expression in a concentration-dependent manner. To clarify whether CDK1 or CDK2 contributes to cell proliferation, *CDK1-* or *CDK2-*specific siRNA was transfected into HeLa cells, and cell proliferative ability was analyzed. In cell lines with siRNA-mediated knockdown of *CDK1*, the number of viable cells was decreased, which was correlated with the growth inhibitory effect. In contrast, no correlation was observed between decreased *CDK2* expression and growth inhibition (Fig. 4e,f).

The cell cycle of HeLa and SiHa cells treated with 9-oxo-ODAs was further evaluated using propidium iodide (PI). The analysis revealed that 9-oxo-ODAs induced cell cycle arrest in cervical cancer cell lines based on an increase in the fraction of cells in the G0-G1 phase and decrease in the fraction of cells in the S and G2-M phases (Fig. 4g). Thus, 9-oxo-ODAs can affect the cell cycle via downregulation of CDKs, especially CDK1.

### 9-oxo-ODAs suppress HPV oncoproteins, decreases CDK1 expression, and arrests the cell cycle

**Figure 5:**
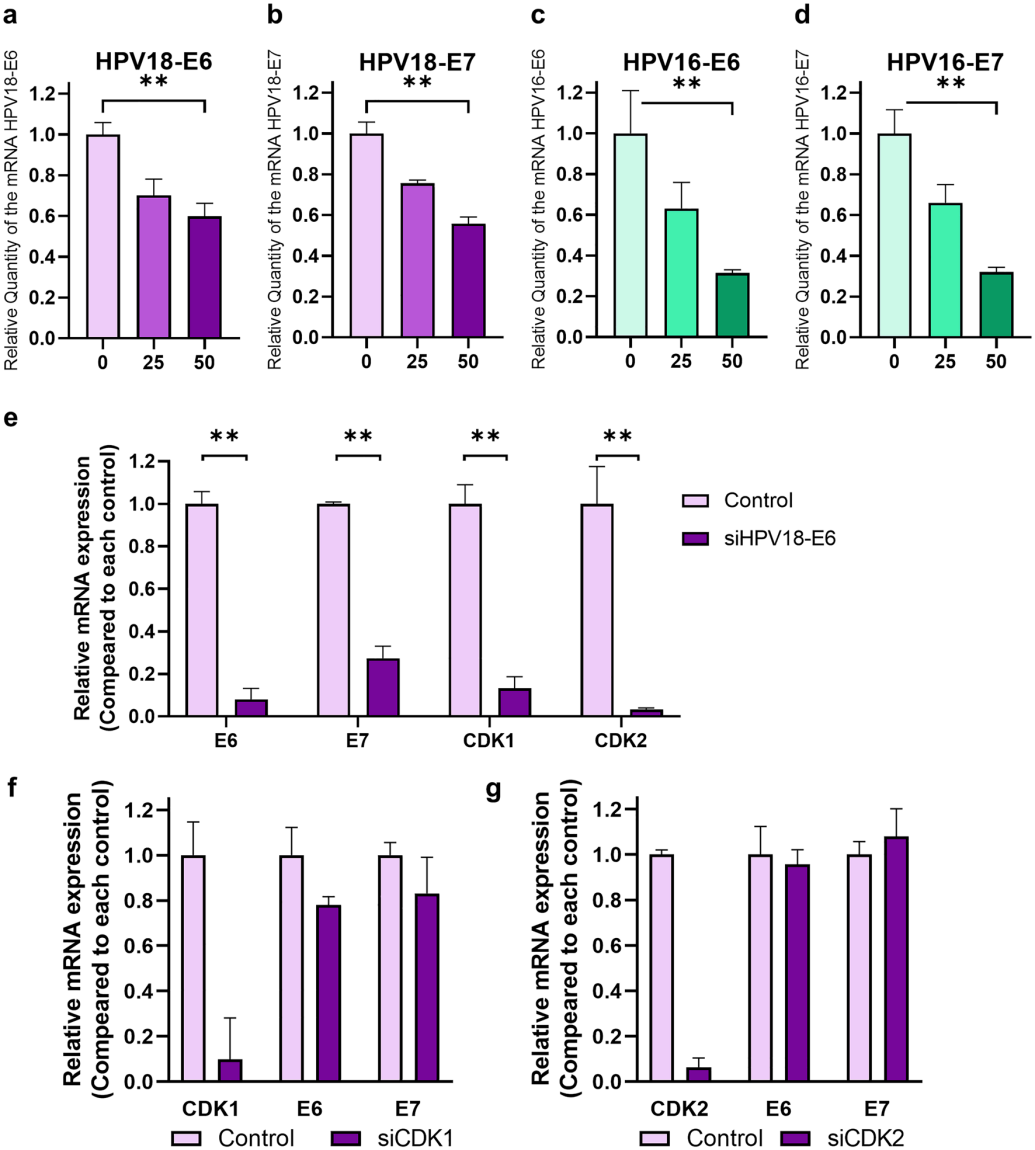
Effects of 9-oxo-ODA treatment on HPV oncoprotein expression and downstream CDK1 and CDK2 expression. 9-oxo-ODAs suppress HPV oncoproteins, including (**a**) HPV16-E6, (**b**) HPV16-E7, (**c**) HPV18-E6, and (**d**) HPV18-E7 in a concentration-dependent manner. (**e**) siRNA-mediated knockdown of E6 reduces mRNA expression of *E6*, *E7*, *CDK1*, and *CDK2*. (**f**) siRNA-mediated knockdown of CDK1 does not affect mRNA expression of E6 and E7. (**g**) siRNA-mediated knockdown of CDK2 does not affect mRNA expression of E6 and E7.

HPV oncoproteins are associated with apoptosis and cell cycle via regulation of p53 and Rb degradation^20,21^. Therefore, we examined whether HPV oncoprotein knockdown would lead to a decrease in CDK1. HPV-E6 and -E7 are oncoproteins that contribute to tumor progression. We analyzed the effects of 9-oxo-ODA treatment on E6 and E7 expression. We observed that 9-oxo-ODAs decreased *E6* and *E7* mRNA expression in a concentration-dependent manner (Fig. 5a–d). To examine the effects of reduced *E6* expression in HeLa cells, HeLa cells were transfected with HPV18-E6-siRNA, and suppression of *E6* expression resulted in reduced *CDK1* and *CDK2* expression (Fig. 5e). HPV18-E6-siRNA downregulated not only *E6* but also *E7* expression, and silencing *E6* alone was difficult even with several candidate primers (Fig. S3). Therefore, downregulation of E6-AP, which functions by forming a complex with E6, using siRNA resulted in a decrease in the number of HeLa cells (Fig. S4). On the other hand, siE6-AP slightly decreased *CDK1* expression and increased *CDK2* expression. These results indicate that the antitumor effect of 9-oxo-ODA involves E6 pathway, but the change in CDK1/CDK2 expression may be more related to E7 pathway. Furthermore, HeLa cells were transfected with CDK1- or CDK2-siRNA, and suppression of *CDK1* or *CDK2* expression did not alter HPV18-*E6* and *E7* expression (Fig. 5f,g). These results indicated that 9-oxo-ODAs may be involved in downregulating HPV oncoprotein expression and leading to cell cycle arrest and apoptosis.

### 9-oxo-ODAs suppress CDK1 expression ex vivo and inhibit metastasis formation in vivo

**Figure 6:**
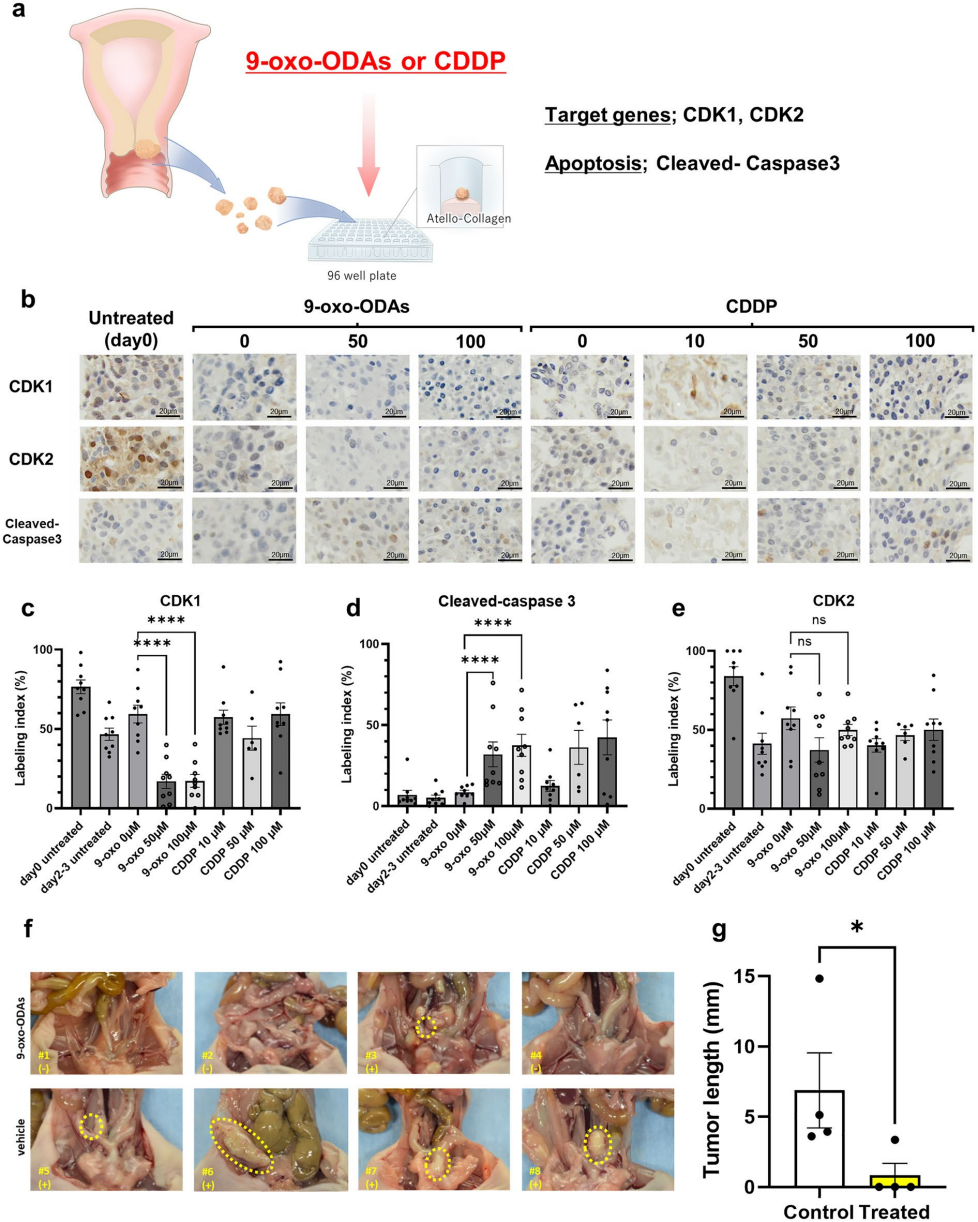
Antitumor effects of 9-oxo-ODAs on HeLa cells ex vivo and in vivo. (**a**) Schematic diagram of tissue collection from cervical cancer clinical specimens and ex vivo assay. (**b**) Representative immunohistochemistry images of CDK1, CDK2, and cleaved-caspase 3 in cervical cancer specimens treated with 9-oxo-ODAs or CDDP. (**c-e**) Positive rates of immunohistochemical staining for (**c**) CDK1, (**d**) cleaved-caspase 3, and (**e**) CDK2 (n = 9). Cutoff values were set for each and evaluated using QuPath. Due to missing data, n = 6 for CDDP 50 μM. (**f**) For in vivo analysis, HeLa-ip4 cells were inoculated into the pelvic cavity of mice followed by treatment with 9-oxo-ODAs or vehicle. (**g**) Comparison of tumor length diameter in 9-oxo-ODAs and vehicle groups.

We then performed ex vivo assays to examine the effects of 9-oxo-ODAs. Clinical samples obtained from human cervical cancer cells were treated with 9-oxo-ODAs or cisplatin (Fig. 6a). The cells were fixed in formalin and immunostained with anti-CDK1, anti-CDK2, and anti-cleaved-caspase 3 antibodies. The results were evaluated using Qupath software (Fig. 6b). Administration of 9-oxo-ODAs decreased CDK1 staining positivity (Fig. 6c) and increased immunopositivity for cleaved-caspase 3, a marker of apoptosis (Fig. 6d). However, there was no significant effect on CDK2 expression (Fig. 6e). This indicated that treatment with 9-oxo-ODAs increased cancer cell apoptosis and decreased CDK1 protein expression ex vivo.

Subsequently, we used an in vivo mouse model in which HeLa cells were injected into the pelvic cavity of mice. Mice were intraperitoneally injected with 9-oxo-ODAs or vehicle 4 weeks after IP, and antitumor effects on metastatic formation in HeLa cells were evaluated. In the group treated with 9-oxo-ODAs, metastatic formation was inhibited, and tumor size was significantly suppressed (Fig. 6f,g).

## Discussion

With the development of vaccines, the prevalence of HPV-associated diseases has increased globally. High-risk HPV types underscore almost all cervical cancer cases as well as a proportion of cancers of the vulva, vagina, penis, anus, and head and neck^22^. In 2012, approximately 4.5% (640,000 cases) of new cancer cases were attributable to HPV infection. Indeed, HPV infection accounted for 29.5% of infection-related cancers worldwide in 2012 and caused more than half of all infection-attributable cancers in women (570,000 cases), with cervical cancer accounting for more than 80% of cases^22^. Therefore, there is an urgent need to develop treatments for HPV-related diseases.

Cervical cancer is caused by HPV, which leads to invasive cancer via cervical dysplasia. Cervical dysplasia results in cervical cancer over several years or decades. Nevertheless, the efficacy of vaccination in patients already infected with HPV remains unknown^23^. Treatment includes close observation with HPV testing, cervical cytology, colposcopy, conization, or ablation of the cervical transformation zone. The efficacy of medical therapies such as imiquimod and 5-fluorouracil (5-FU) has been evaluated as alternative or additional therapies to these conventional therapies; however, trials have been small, limited, and impractical^24–27^. The topical use of imiquimod is effective in patients with cervical intraepithelial neoplasia (CIN) 2–3 but frequently causes vulvar pain, pruritus, headache, myalgia, and flu-like symptoms^24^. Other potential agents, such as retinoids, interferons, antivirals, and hormonal therapy, have also been evaluated, but there is a paucity of data in this regard^28,29^. In contrast, 9-oxo-ODAs, a natural compound, lack any obvious toxicity and did not significantly alter viability against human primary mesothelial cell (HPMC) used as a control in this study. Therefore, 9-oxo-ODAs may be considered safe for application on skin and mucosal sites, highlighting their clinical applicability.

To summarize our results, 9-oxo-ODAs are expected to inhibit cell proliferation by suppressing the expression of CDK1 and HPV oncoproteins, arresting and reducing the cell cycle of cervical cancer, and inducing partial apoptosis. Previous studies have suggested that specific siRNAs targeting the *E6* and *E7* genes of HPV16 or HPV18 upregulate p53 and Rb and induce apoptosis^30,31^. In this study, 9-oxo-ODAs downregulated the expression of E6 and E7 in cervical cancer cells, slowed the cell cycle, and induced apoptosis. Previous reports have demonstrated that suppression of HPV oncoprotein expression causes cell cycle inhibition^31,32^. Our results indicated that the antitumor effects of 9-oxo-ODAs may involve the E6 and E7 pathway. However, the mechanisms by which 9-oxo-ODAs downregulate E6 and E7 expression remain unknown. 9-oxo-ODAs are speculated to act on the promoter regions and transcription factors of the *E6* and *E7* genes. Although decreased expression of CDKs may delay the cell cycle, it is unclear whether this is a direct effect of 9-oxo-ODAs or a phenomenon associated with cell cycle inhibition, which should be clarified in future research.

9-oxo-ODAs are potent PPARα ligands that ameliorate abnormalities in lipid metabolism^33,34^. PPARα regulates SMC cell cycle progression at the G1/S transition by targeting the CDK inhibitor and tumor suppressor p16INK4a (p16), resulting in inhibition of retinoblastoma protein phosphorylation. Moreover, PPARα activation inhibits SMC growth in vivo, an effect dependent on p16 expression^35^. Further, PPARα activators stimulate differentiation, inhibit proliferation, and increase apoptosis in cultured human keratinocytes^36^. Collectively, these findings suggest that 9-oxo-ODAs may suppress the proliferation of cervical cancer cells and induce apoptosis via PPARα, but the underlying mechanisms warrant further clarification.

High-risk types of carcinogenic HPVs include types 16 and 18, as well as types 31, 33, 35, 45, 52, and 58, which are detected at high frequencies, although there are regional differences^37^. In Japan, types 16 and 18 are slightly less common, while types 52 and 58 tend to be found in a higher percentage of cancer tissues^38^. The data in this study focused on cell lines for HPV types 16 and 18; hence, it is necessary to examine the effects of 9-oxo-ODAs on other subtypes. It has been suggested that 9-oxo-ODAs extracted from eggplant exhibit antitumor effects by suppressing the expression of CDK1 and HPV oncoproteins, E6 and E7, in cervical cancer cells and tissues, in addition to inducing tumor-specific cell cycle arrest and apoptosis.

In conclusion, 9-oxo-ODAs, which are a component of the calyx of eggplant, can induce growth inhibition in cervical cancer cells both in vitro and in vivo. These effects may be due to decreased expression of HPV oncoprotein E6/E7 and consequent suppression of CDK1 expression. Our findings highlight 9-oxo-ODAs as promising therapeutic candidates for the treatment of HPV-positive diseases.

## Methods

### Cell lines

Human cervical cancer cell lines (HeLa and SiHa) were grown in RPMI medium (SIGMA, Osaka, Japan) supplemented with heat-inactivated fetal bovine serum (FBS; Thermo Fisher Scientific, Yokohama, Japan) and 1% penicillin–streptomycin (Nacalai Tesque, Kyoto, Japan). These cell lines were purchased from the American Type Culture Collection (Manassas, Virginia, USA) and were authenticated by short tandem repeat profiling (BEX, Tokyo, Japan). HeLa-ip4 cells were established by inoculating HeLa cells into the abdominal cavity of nude mice and passaging them four times in individual mice. Human oral squamous cell carcinoma cell lines (HSC-2, SCC-25 and SAS) were grown in RPMI medium (SIGMA, Osaka, Japan) supplemented with heat-inactivated fetal bovine serum (FBS; Thermo Fisher Scientific, Yokohama, Japan) and 1% penicillin–streptomycin (Nacalai Tesque, Kyoto, Japan). HSC-2 and SAS were obtained from the Cell Resource Center for Biomedical Research (Tohoku University, Miyagi, Japan), and SCC-25 was were purchased from the American Type Culture Collection (Manassas, Virginia, USA). Human primary mesothelial cells (HPMCs) were isolated^39^ from the tumor-free omentum of patients with malignant ovarian tumors. HPMCs were cultured on collagen-coated plates in RPMI-1640 medium supplemented with 10% FBS and penicillin/streptomycin. Cells were cultured at 37 °C in a humidified incubator with 5% CO_2_, their passage number was within 30 times.

### Reagents

The calyx of eggplant was minced (1300 g) and air-dried (100 g), followed by the addition of 98% ethanol (500 mL) for extraction at 15–25 °C. The extract was concentrated thrice for testing. Experimental research and field studies on plants (either cultivated or wild), including the collection of plant material have complied with relevant institutional, national, and international guidelines and legislation. 9-oxo-OAs, comprising 9-oxo-(10E,12Z)-octadecadienoic acid and 9-oxo-(10E,12E)-octadecadienoic acid, were purchased from Shinsei Chemical Company Ltd. (Osaka, Japan).

### In vitro proliferation assay

In total, 10^3^ cells of the various cell lines were cultured in 96-well plates, and a dilution series of 9-oxo-ODAs was added to the cultured cells the next day. After 3–5 days, cell viability was determined using a Cell Counting Kit-8 (DOJINDO, Kumamoto, Japan). Briefly, 10 μL of Cell Counting solution was added to each well, and the plate was incubated at 37 °C for 3 h. The absorbance at 450 nm was measured. The trypan blue exclusion method was used to measure living cells. Briefly, 10^5^ cells of the various cell lines were cultured in 6-well plates, and a dilution series of 9-oxo-ODAs was added to the cultured cells the next day. Living cells were measured using a Vi-Cell XR (Beckman Coulter Life Sciences, Tokyo, Japan).

### Immunoblotting assay

Immunoblot analysis was performed to detect various antigens in the clinical samples and cell lines, as described previously^40^. The blots were cut prior to hybridization with antibodies, and the blots are presented in Supplementary Fig. 2. The following antibodies (Abs) were utilized: anti-CDK1 polyclonal antibody (catalog 19532–1-AP, Proteintech, Rosemont, USA), anti-CDK2 monoclonal antibody (catalog ab32147, Abcam, Cambridge, UK), and anti-GAPDH monoclonal antibody (Cell Signaling Technology, Beverly, MA, USA).

### Quantification of mRNA expression levels

We used a semi-quantitative PCR method to detect mRNAs in human ovarian cancer cell lines and in the ovarian surface epithelium. Human ovarian surface epithelial cells were purchased from Cosmo Bio. Total RNA was extracted from cell lines using the RNeasy Mini Kit (Qiagen) and subjected to qPCR. cDNA was synthesized from 1 µg of total RNA using a High-Capacity cDNA Reverse Transcription Kit (Applied Biosystems) with oligo-dT primers. Fast SYBER Green Master Mix (Applied Biosystems) was used for amplification, and the samples were run on a Step One system (Applied Biosystems). Primers were designed to detect spliced mRNA, and their sequences are presented in Supplementary Table 1. All primers were synthesized and purchased from Hokkaido System Science.

### RNA interference and transfections

siRNAs were synthesized and purchased from Hokkaido System Science (Sapporo, Japan) and are listed in Supplementary Table 2. siRNA against HPV18-E6 and E6-AP were purchased from Santa Cruz Biotechnology (Dallas, TX, USA). AllStars Negative Control siRNA (Qiagen) Tokyo, Japan) was used as a negative control. All siRNA transfections were performed using Lipofectamine RNAiMAX (Thermo Fisher Scientific, Waltham, MA, USA).

### RNA sequence analysis

Samples were prepared as described in our previous report^41^. For RNA sequence analysis (Fig. 2), HeLa cells were treated with vehicle (DMSO) or 9-oxo-ODAs (50 μM) for 72 h. Total RNA was isolated using the RNeasy mini kit (Qiagen). RNA integrity was confirmed using a NanoDrop spectrophotometer and Agilent Bioanalyzer 2100. Sequencing was performed using NovaSeq 6000 instrument (Illumina, CA, USA). We mapped the reads to a reference genome (Human, NCBI GRCh38) using Hisat2 (version 2.2.0) with default settings^42^. Relative gene expression levels were estimated by fragments per kilobase of transcript per million mapped reads using Stringtie (version 2.1.1) with default settings^43^. We used Genespring (version 14.9, Agilent) for expression analysis. To minimize noise, genes with low expression levels were filtered out by applying a minimum percentile of 20%. Using a bioinformatics web tool, the database for annotation, visualization, and integrated discovery (DAVID, http://david.abcc.ncifcrf.gov, and version 6.8), we performed KEGG pathway analysis and converted gene ID to protein ID. GSEA was performed using the desktop application (http://www.broadinstitute.org/gsea/index.jsp). Gene sets were acquired from the Molecular Signatures Database, including HALLMARK_E2F_TARGETS (M5925), HALLMARK_G2M_CHECKPOINT (M5901), HALLMARK_P53_PATHWAY (M5939), and HALLMARK_APOPTOSIS (M59023).

### Mass spectrometry

HeLa and SiHa cells were plated 10^5^ cells per well and 9-oxo-ODAs were added to the culture media (final concentration: 0 mM, 25 mM, and 50 mM). After culturing for 72 h, samples were prepared from these cells and the label free quantification of proteins were conducted according to our previous report^44^. Log-transformed and median-centered values were used and proteins without missing values in each dataset (HeLa or SiHa) were further analyzed. For heatmap depiction, venn diagram generation, and gene ontology (GO) enrichment analysis, following softwares were used: R (V4.1.1), RStudio (2021.09.0), pheatmap package (V1.0.12)^45^, gplots package (V3.1.3)^46^, clusterProfiler package (V4.0.5)^47,48^.

### Flow cytometry analysis

10^5^ cells of the various cell lines were cultured in 6-well plates, and a dilution series of 9-oxo-ODAs was added to the cultured cells the next day. Apoptosis was analyzed using an APC Annexin V Apoptosis Detection Kit with 7-AAD (BioLegend, San Diego, CA) on a FACS Aria II (BD Biosciences, Franklin Lakes, NJ, USA). Cell cycle analysis was performed using nuclear staining with propidium iodide (SIGMA, St. Louis, MO, USA) and FACS Aria II.

### Ex vivo and immunohistochemistry

Cervical cancer tissue samples (n = 3) from patients were collected prospectively from 2020 to 2021 at Nagoya University Hospital, in accordance with the guidelines established by the Ethics Committee of Nagoya University. This study was approved by the Ethics Committee of Nagoya University (trial number: 2017-0497). Informed consent was obtained from all subjects. Clinical information on the samples is shown in Supplementary Table 3. The collected samples were stored in STEM-CELLBANKER GMP grade (catalog CB047, TAKARA) and frozen at − 80 °C. Sample processing was performed as reported previously^49^. After thawing, the tissue was cut into 1 mm3 pieces using a scalpel, and 3–4 pieces/sponge for each treatment were explanted onto AteloCell (catalog CSH-96, KAKEN) in 96-well plates immersed in 70–100 μL of RPMI-1640 media supplemented with 10% FBS and antibiotics in addition to increasing concentrations of cisplatin (0–100 μM) or 9-oxo-ODAs (0–100 μM, dissolved in DMSO, Shinsei Chemical Company Ltd.) in a humidified atmosphere at 37 °C containing 5% CO_2_. Tissues were harvested after 48–72 h and fixed with 4% buffered formalin.

Sections from paraffin-embedded tissue blocks (4-µm thick) were probed with anti-CDK1 polyclonal antibody (Proteintech, Rosemont, USA,1:100), anti-CDK2 monoclonal antibody (Abcam, Cambridge, UK, 1:50), and anti-cleaved caspase 3 polyclonal antibody (Cell Signaling Technology, Beverly, MA, USA, 1:100). A blocking buffer without the primary antibody was used as a negative control. Sample images were captured using a VS120. Three sites containing 100–1000 cells were arbitrarily selected from each sample image, and the labeling index was evaluated using QuPath^50^.

### Animals and animal experiments

The animal experimental protocol was approved by Nagoya University (approval number: M230302-005, M230303-004), and all experiments were conducted in accordance with relevant guidelines and regulations. The study was carried out in compliance with the ARRIVE guidelines. Inbred nude CAnN.Cg-Foxn1^nu^/CrlCrlj (nude) mice were purchased from Charles River Laboratories International (Yokohama, Japan). Mice were maintained under specific pathogen-free conditions in the Division of Experimental Animals, Nagoya University. Eight-week-old nude mice were intraperitoneally inoculated with HeLa-ip4 (5 × 10^5^ cells/animal). The next day, 9-oxo-ODAs (100 μL of PBS(−) with 30 μg/10 μL in ethanol) or vehicle (100 μL of PBS(−) with 10 μL of ethanol) were administered intraperitoneally once a week. After 8 weeks, the mice were euthanized by carbon dioxide (CO_2_) overdose for observation.

### Statistical methods

Statistical analysis was performed using GraphPad Prism 9 (V9.4.0) software. The Mann–Whitney U test was used to assess statistical significance between the control and treatment groups. Data are presented as the mean and standard error of the mean. Statistical significance was set at P < 0.05.

### Supplementary Information

Supplementary Figure 1.

Supplementary Figure 2.

Supplementary Figure 3.

Supplementary Figure 4.

Supplementary Table 1.

Supplementary Table 2.

Supplementary Table 3.

## Data Availability

The data that support the findings of this study are available on request from the corresponding author. The RNA-Seq datasets produced in this study are deposited in the DDBJ (PRJDB14062). The mass spectrometry proteomics data have been deposited to the ProteomeXchange Consortium via the PRIDE partner repository with the dataset identifier PXD036713 and https://doi.org/10.6019/PXD036713.
