# Supplementary figures and images for: 9-oxo-ODAs suppresses the proliferation of human cervical cancer cells through the inhibition of CDKs and HPV oncoproteins
Source: Sci Rep. 2023 Nov 6;13:19208. doi: 10.1038/s41598-023-44365-3 (PMC10628276; doi:10.1038/s41598-023-44365-3)

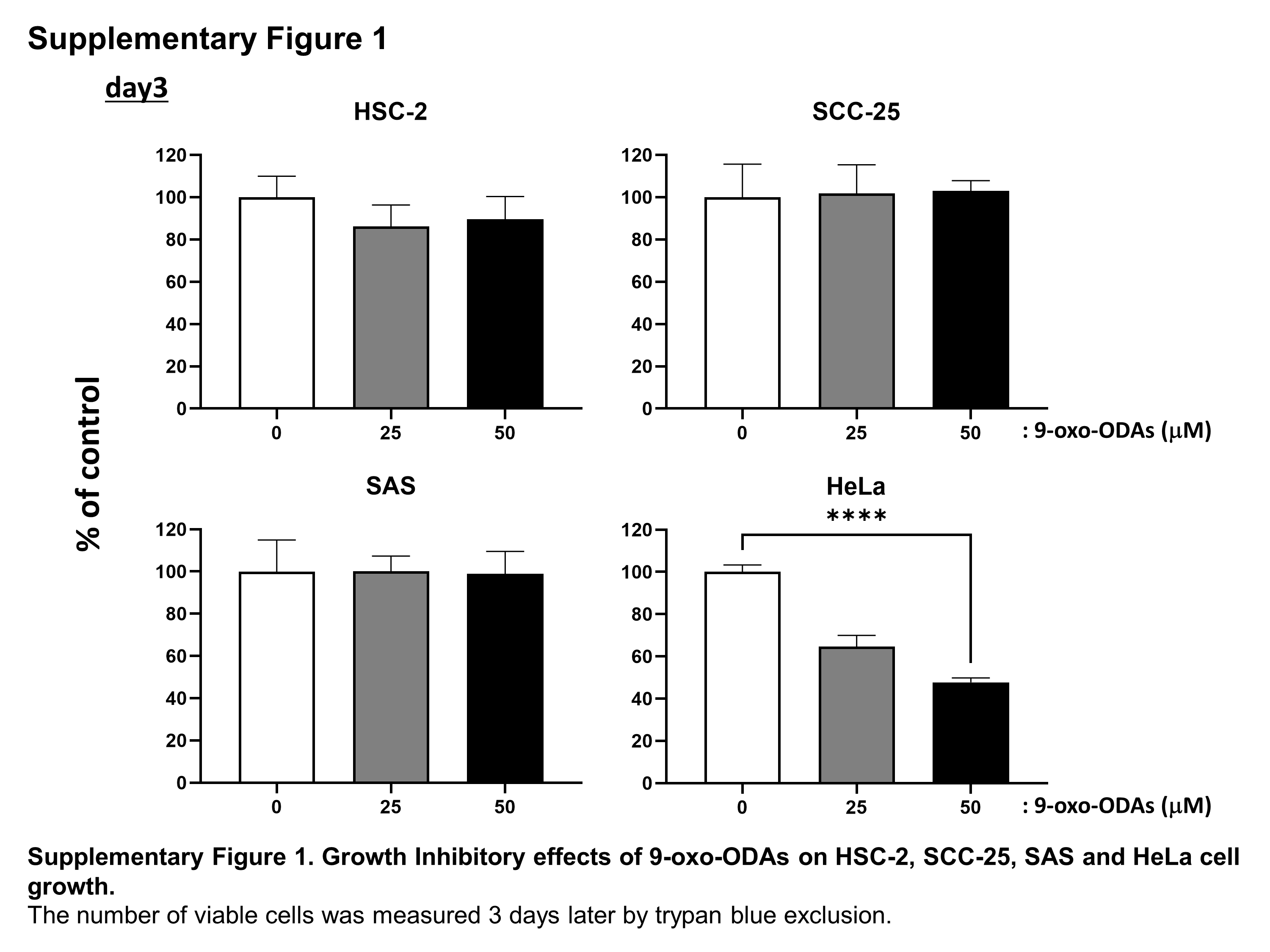

Supplement: Supplementary file 1 — Supplementary Figure 1. [file 41598_2023_44365_MOESM1_ESM.tif]

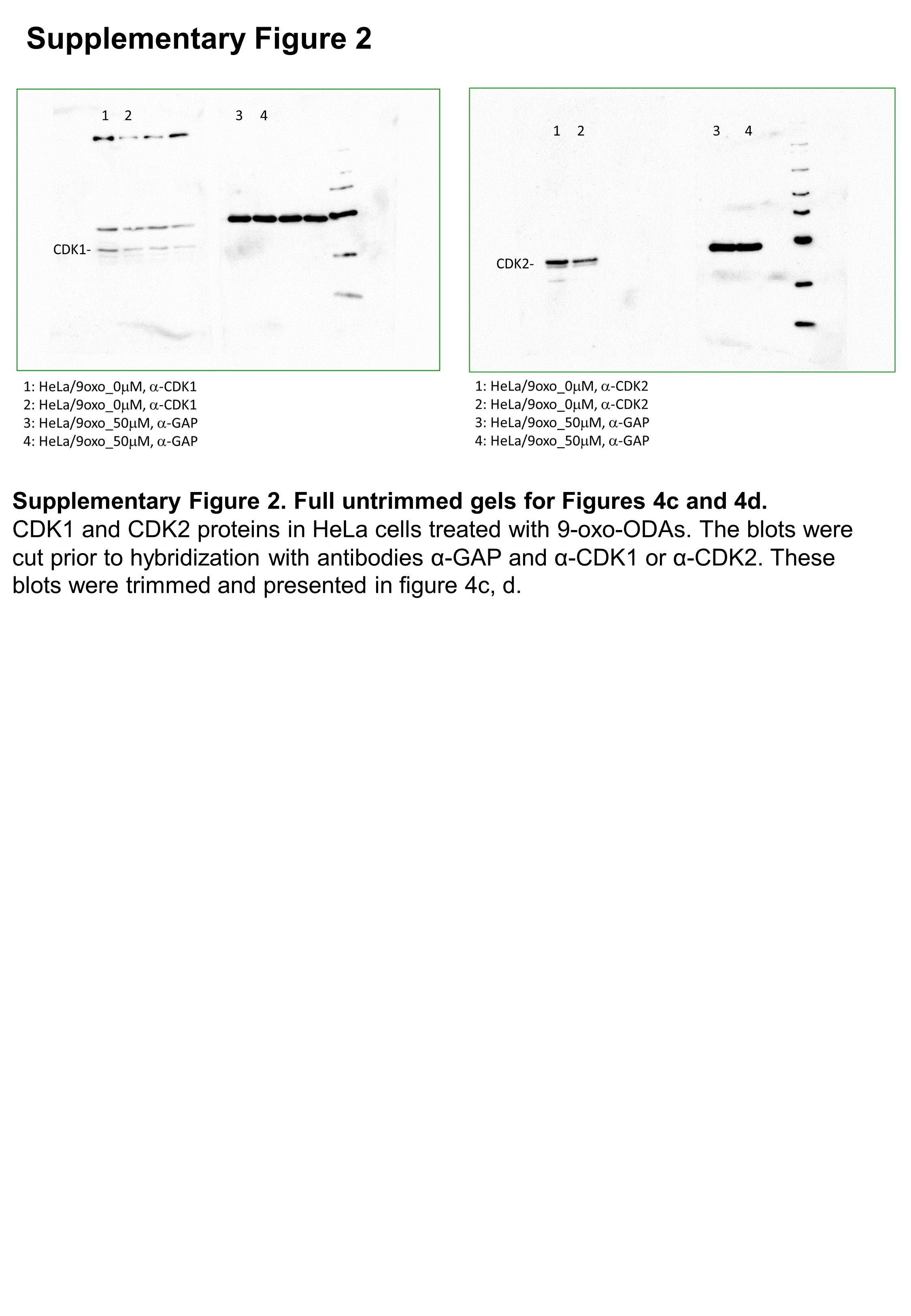

Supplement: Supplementary file 2 — Supplementary Figure 2. [file 41598_2023_44365_MOESM2_ESM.tif]

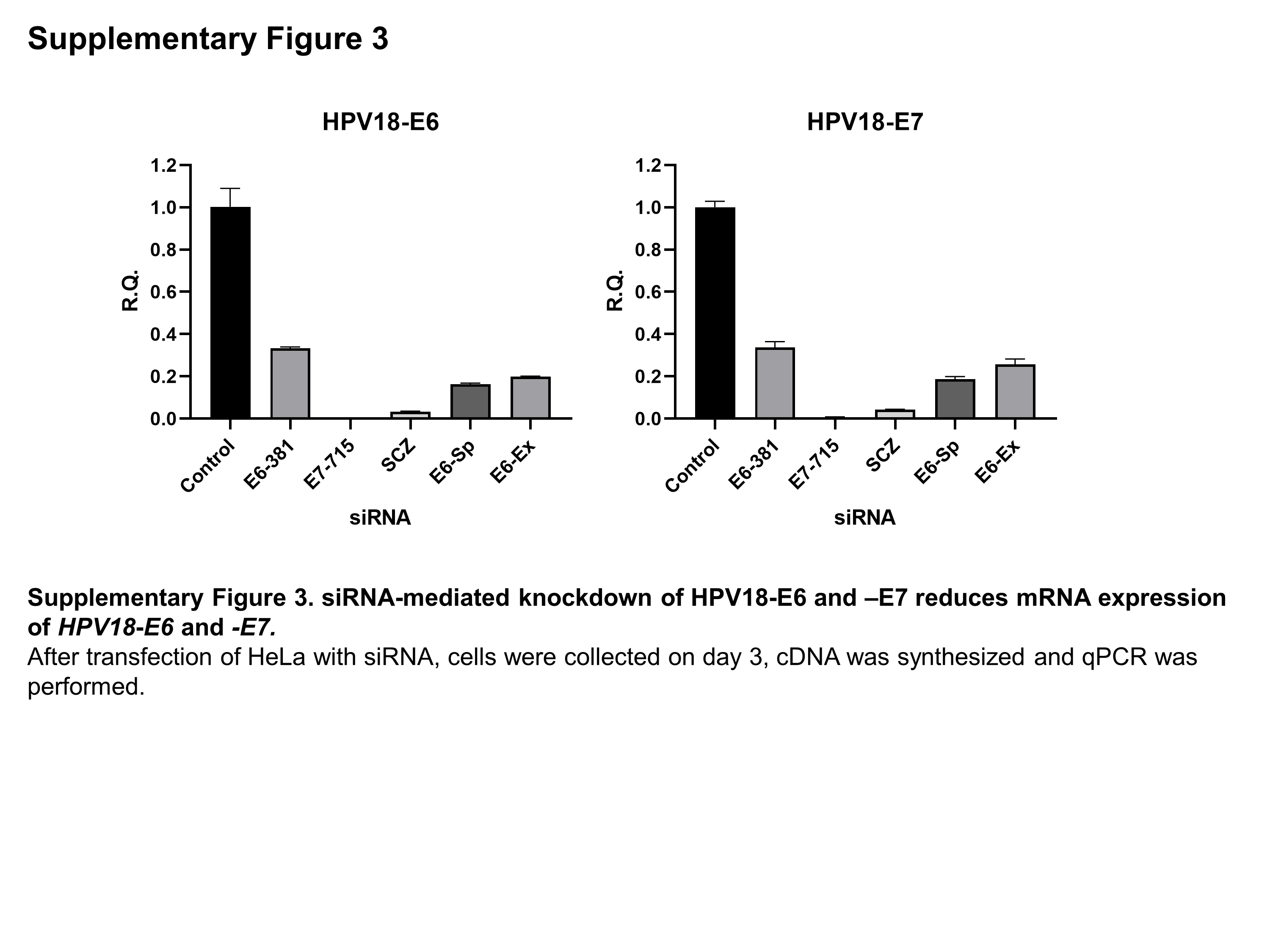

Supplement: Supplementary file 3 — Supplementary Figure 3. [file 41598_2023_44365_MOESM3_ESM.tif]

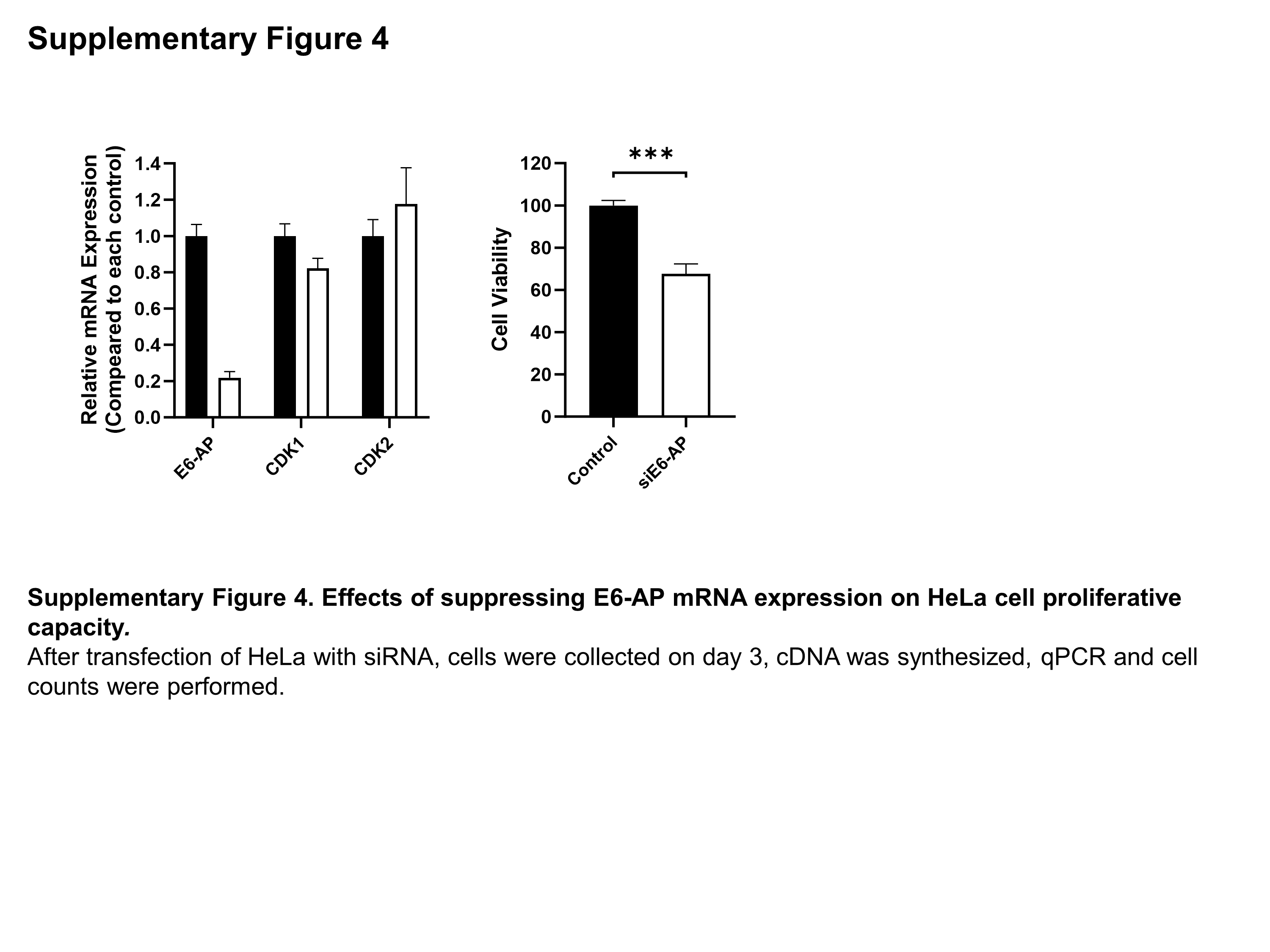

Supplement: Supplementary file 4 — Supplementary Figure 4. [file 41598_2023_44365_MOESM4_ESM.tif]
